# Effects of Fish Oil Replacement With Degossypolized Cottonseed Oil on Growth Performance, Proximate Composition, Lipid Metabolism, and Liver Function of Large Yellow Croaker (*Larimichthys crocea*) Juvenile

**DOI:** 10.1155/anu/4241105

**Published:** 2025-06-13

**Authors:** Md. Golam Sajed Riar, Nur A Raushon, Shijie Pan, Jinze Zhang, Chukwuma Kenneth Chibuikem, Yueru Li, Kangsen Mai, Qinghui Ai

**Affiliations:** ^1^Key laboratory of Aquaculture Nutrition and Feed, Ministry of Agriculture and Rural Affairs, and the Key Laboratory of Mariculture, Ministry of Education, Ocean University of China, Department of Aquaculture, Qingdao 266003, Shandong, China; ^2^Bangladesh Fisheries Research Institute, Headquarters, Mymensingh, Dhaka 2201, Bangladesh; ^3^Laboratory for Marine Fisheries Science and Food Production Processes, Qingdao Marine Science and Technology, Qingdao 266237, Shandong, China

**Keywords:** degossypolized cottonseed oil, growth performance, *Larimichthys crocea*, lipid metabolism, liver function

## Abstract

A 10-week feeding trial was conducted with juvenile large yellow croaker, *Larimichthys crocea*, to evaluate the effects of degossypolized cottonseed oil (DCSO) as a potential replacement for fish oil (FO). Five isonitrogenous (42% crude protein) and isolipidic (13% crude fat) diets were formulated where DCSO replaced FO at 0%, 25%, 50%, 75%, and 100% (DCSO0, DCSO25, DCSO50, DCSO75, and DCSO100) to determine their effects on growth performance, feed utilization and tissue lipid compositions of experimental fish. The initial body weight of the experimental fish was 8.28 ± 0.99 g. Results showed that substituting up to 50% of FO with DCSO does not significantly affect the fish's survival rate (SR), growth performance, or proximate composition. However, exceeding a 50% replacement significantly decreases (*p* < 0.05) growth performance and feed efficiency. Additionally, significant increases (*p* < 0.05) in viscerosomatic index (VSI) and hepatosomatic index (HSI) are observed at higher DCSO inclusion levels. Fatty acid analysis shows that DCSO is rich in *n*-6 polyunsaturated fatty acids (PUFA). Still, low in *n*-3 PUFA alters fatty acid profiles in the liver and muscle, resulting in elevated total cholesterol (T-CHO), triglycerides (TG), and low-density lipoprotein cholesterol (LDL-C) levels, alongside increased activities of liver enzymes, indicating compromised liver function. While antioxidant enzyme levels remain stable, malondialdehyde (MDA) levels rise, suggesting elevated oxidative stress. Furthermore, lipase activity declines significantly (*p* < 0.05) at 50% FO replacement, with upregulated lipid synthesis genes (*srebp1*, *fatp1*, *fas*, and *ppar-α*) leading to higher lipid accumulation. Inflammatory response gene expression is also significantly affected, showing amplified pro-inflammatory (*ifnγ*, *il-1β*, and *tnfα*) and decreased anti-inflammatory (*il-10*, *arg-1*, *tgfβ*) cytokines at higher DCSO levels. In conclusion, while up to 50% replacement of FO with DCSO is acceptable, surpassing this threshold adversely affects growth, liver function, lipid metabolism, and inflammatory responses in juvenile large yellow croaker.

**Summary**

• Fish oil (FO) replacement by degossypolized cottonseed oil (DCSO) up to 50% did not negatively affect growth performance and feed utilization in large yellow croaker.
• The patterns of fatty acid profiles in both liver and muscle were drastically change in 75% and 100% replacement of fish oil with DCSO in the diets.
• The high inclusion of DCSO in the diets would evoke liver inflammation in large yellow croaker.
• The unbalanced fatty acid profiles of DCSO aggravated the lipid accumulation in the liver and impaired liver functions.

## 1. Introduction

Balanced fatty acids profiles and abundance in *n*-3 long-chain polyunsaturated fatty acids (LC-PUFA)s contents along with high levels of fat-soluble vitamins, fish oil (FO) is regarded as the most suitable lipid source in the formulation of commercial aquafeeds [1, 2]. However, with the rapid expansion of the aquaculture industry, the decreasing availability and increasing cost of FO in recent years have limited the utilization of FO in aquafeeds [3]. Therefore, the exploitation of other lipid source as alternatives and evaluation their effects in fish growth, feed utilization, immunity, as well as quality are a subject of active investigation in aquaculture [4, 5]. Owing to the cost-effectiveness, wide availability and abundance of unsaturated fatty acids, the research on plant-based lipid sources has gained prevalence, among others. Furthermore, recent studies indicated that in certain fish species and farming stages, FO can be replaced with plant-based lipid sources such as rapeseed oil [6] and sunflower oil [7] in *Oncorhynchus mykiss*, linseed oil in *Brachymystax lenok* [8], soybean oil in *Larimichthys crocea* [9], germ oil in juvenile hybrid grouper (*Epinephelus fuscoguttatus*♀ × *Epinephelus lanceolatus*♂) [10], palm oil in *Thamnaconus septentrionalis* [11] providing a promising strategy for sustainable development of aquaculture industry.

Although certain plant-based lipids could benefit the health status of farmed fish in the aspects of promoting anti-oxidant and anti-inflammation [12], their applications in entirely substituting FO in aquafeed of marine fish have several significant limitations. These lipids share the universal scarcity features in *n*-3 PUFA, docosahexaenoic acid (DHA) and eicosapentaenoic acid (EPA) and the unbalanced fatty profiles and potential residues of anti-nutritional compounds [13, 14]. Compared to freshwater fish, the limited *n*-3 PUFA synthetic capacity of marine fish leads to their higher requirement of these essential fatty acids [15, 16]. Therefore, a complete replacement of FO with plant-based lipids will cause inadequate *n*-3 PUFA in the fish diets and ultimately affect the fish's growth performances and survival rates (SR). Meanwhile, the deficiency in *n*-3 PUFA is associated with fatty liver disease, evidenced by the excessive amount of plant oil in fish feed leading to excess lipid accumulation in the liver of fish [17]. Furthermore, the unbalanced fatty profile in plant-based oil may also contribute to fatty liver disease in fish. The saturated fatty acids (SFA), confirmed as a primary contributor to the progression of fatty liver disease in mammals [18], are enriched in plant-based lipids such as palm oil and coconut oil. Thus, understanding the features of the fatty acid profile in plant-based lipids and designing appropriate replacement levels provide a feasible strategy that benefits mariculture economically and sustainably.

Degossypolized cottonseed oil (DCSO) is a plant-based lipid extracted from the cotton plant's seeds [19]. The abundance of essential fatty acids (especially in linoleic acid) together with active components such as vitamin E and phytosterol make DCSO an ideal lipid source in both livestock feeds [20], broilers [21] and aquafeeds [22, 23]. In aquafeeds, although studies have shown that DCSO could partially replace FO as a dietary lipid source in several fish species in terms of feed utilization [22, 23] and growth performance [24, 25], higher inclusion of DCSO always leads to the increase of viscerosomatic index (VSI) and hepatosomatic index (HSI) of the European Sea Bass, *Dicentrarchus labrax* [22]. Besides, gossypol, which is an anti-nutritive compound derived from pigment glands in cotton seed and residue in DCSO, could cause detrimental effects such as impaired body weight gain, anorexia, weakness, and even death the animals [26]. Thus, the removal of gossypol residue and limiting its concentration to a very low range by using solvents and refining techniques [27, 28] are the initial steps to reduce the detrimental effects and promote the applications of DCSO in animal nutrition. So far, no gossypol (less than 1μg/kg) was detectable in commercial edible DCSO [29]. To date, limited studies have been reported about the effect of DCSO as a substitute for FO in aquafeeds; many potential substitution effects remain unknown, so further studies are urgently needed.

Large yellow croaker is one of the most profitable marine fish species, with the highest yearly production among the farmed marine fish in China [30]. The shortage of FO sources has boosted the extensive studies on the effects of replacing FO with different plant-based lipids on large yellow croaker, like soybean oil [31], olive oil [17]; coconut oil [32]; and rice bran oil [33]. However, in this marine species, the metabolic processes are easily influenced by the composition of dietary fatty acids, evident by the excess replacement of FO by these plant-based lipids aggravated the lipid accumulation and altered fatty acids profiles in the tissues in large yellow croaker juvenile in both terrestrial oil [34, 35] and vegetable oil [33]. Until now, no studies have been conducted on the partial or total replacement of FO with DCSO in the nutrition of large yellow croaker. Therefore, this study was conducted to provide a theoretical basis for applying DCSO in large yellow croaker juvenile feed and determine its effects on growth performance, feed utilization, lipid metabolism and inflammation. The Results of this study will provide a theoretical basis for applyingDCSO in large yellow croaker juvenile feed.

## 2. Materials and Methods

### 2.1. Ethics Statement

All fish handling procedure of this experiment are strictly following the standard protocol of the Animal Care Committee of the Ocean University of China (Approval No. SPXY2020012).

### 2.2. Experimental Plan and Feed Formulation

Five isonitrogenous (42% crude protein) and isolipidic (13% crude fat) diets were formulated, replacing FO with DCSO in the percentage ratio of DCSO 0%_FO 100%, (Control), DCSO 25%_ FO 75%, DCSO 50%_FO 50%, DCSO 75%_FO 25%, and DCSO 100%_FO 0% in this study (Table 1). Experimental edible DCSO (zero gossypol) was obtained from Xinjiang Herun Jinlan Biotechnology Co. Ltd. Dietary protein sources (soybean meal, white fish meal, bread flour, etc.) were initially mashed by hand, mechanically crushed and then sieved into fine powder and then blended with water and choline chloride. The mixture was processed using an automatic pellet-making machine (F-26 (II), South China University of Technology, China), creating hard pellets with dimensions of 4.0 mm x 4.0 mm and 4.0 mm x 6.0 mm. The pellets were oven-dried (CT-C-1, Jiang Yin Zhou Yuan Pharmaceutical Equipment Co., Ltd., Jiangsu, China) for 12 h at 55°C and packed and stored in a refrigerator at − 20°C until trial and analysis.

### 2.3. Experimental Fish and Procedure

The feeding trial was conducted at Xiangshan Bay, Ningbo City, Zhejiang Province, China. Before the experiment, large yellow croaker juveniles were acclimated for 14 days in a floating net cage. Then, 900 healthy fish were randomly assigned to floating cages, with 60 fish (initial body weight of 8.28 ± 0.99 g) per cage (1 m × 1 m × 1 m), with three replicate cages per treatment. Fish were fed to apparent satiation twice a day at 05:00 and 17:00 for 70 days. Water quality and temperature were routinely measured daily by using Professional Plus Multiparameter Instrument The water parameters during the growth trial ranged from 18.9 to 29.5°C, with salinity ranging from 27.5 to 29 g/L, dissolved oxygen approximately 7.0 mg/L, and pH ranging from 7 to 8.0. Ammonia content wasn't considered in open water culture habitat.2.4. Sampling

At the end of the experiment, the fish were fasted for 24 h to empty the gut and then anesthetized by MS222. The number of fish in each cage was counted and weighted to calculate the SR, weight gain rate (WGR) and specific growth rate (SGR). From each cage, five fish were randomly selected, dissected on ice, and weighed to determine the HSI and VSI and five fish from each cage were saved for whole-body composition analysis.

### 2.4. Proximate Composition of Diet and Fish

The proximate composition, including crude protein, crude lipid, and moisture of the samples (fish whole body, liver total lipids and diets) were determined in this study. Briefly, the crude protein content of the fish and feeds was analyzed using the Kjeldahl nitrogen method (FOSS Kjeltec 8400 Analyzer Uni, Sweden). The crude lipid content of the whole fish and feeds was determined using the Soxhlet extraction method (Soxhlet Extraction System B-801, Buchi, Switzerland). The moisture content of the samples was examined by consistent oven-drying at 105°C for 24 h, after which it was allowed to cool before taking the measurement, the determination of crude ash involves subjecting the sample to a muffle furnace operating at a temperature of 550°C, fully burning it to constant weight, and calculating the difference following the official AOAC [36] method. All samples were analyzed in triplicate.

### 2.5. Analysis of Fatty Acid Profiles in Diets and Tissues

To analyze the fatty acid profiles in the diets and fish tissue, the samples (diets, livers and muscles) were initially preserved at −20°C for 24 h and lyophilized for 72 h using the CHRIST Alpha 1–4 LDplus machine and then stored at room temperature. The 100 mg of lyophilized sample was then placed in a 10 mL graduated test tube, and 3 mL of 1N KOH-methanol solution (Regan Biotechnology Co., Ltd., Anhui, China) was added. The mixture was heated in a water bath for 20 min at 75–80°C and neutralized by 3 mL of HCl- methanol (Regan Biotechnology Co., Ltd., Anhui, China). The extract was then agitated with *n*-hexane, settling into distinct layers at room temperature. The upper layer was then separated by centrifuging at 5000 rpm for 5 min at 4°C [37, 38]. The fatty acid methyl esters (FAMEs) were quantified by HP6890 gas chromatograph (Agilents Technologies Inc., Santa Clara, California, USA) with a fuzed silica capillary column (DB-23, 60 m × 0.25 mm, 0.15 μm).

### 2.6. Activities of Intestinal Digestive Enzyme

The intestinal digestive enzyme activities were measured using Nanjing Jiancheng Bioengineering Institute (Nanjing, China) commercial kits. Briefly, the samples of intestinal liquid were collected and thoroughly mixed and ground into a fine powder using a homogenate medium at 1:9 ratios. Then, the liquid was centrifuged at 2500 rpm for 10 min at 4°C to separate the supernatant. The protein content of the sample homogenates was determined using the Coomassie brilliant blue technique and a commercial kit (A054-2-1).

### 2.7. Serum Biochemical Parameters

The serum properties, including the content of low-density lipoprotein cholesterol (LDL-C), high-density lipoprotein cholesterol (HDL-C), total cholesterol (T-CHO), triglycerides (TG), total protein (TP), and the activities of aspartate aminotransferase (AST) and alanine aminotransferase (ALT), were detected immediately after sampling by diagnostic reagent kits (Nanjing Jiancheng Bioengineering Institute, China) following the manufacturer's instructions. Briefly, the concentrations of serum samples were first diluted to measuring range according to the kits; then, the parameters including TP (A045-2), T-CHO (A111-1-1), ALT (C009-2-1), AST (C010-2-1), LDL-C (A113-1-1), HDL-C (A112-1-1)and TG (A110-1-1) were measured separately.

### 2.8. Hepatic and Intestinal Antioxidant Capacity

The antioxidant capacity of the liver and intestine was measured in this study. Briefly, samples from different tissues were collected and crushed in homogenate mediums, followed by homogenizing and centrifuging at 2500 rpm for 10 min at 4°C. After that, protein contents from each sample were measured through the Coomassie brilliant blue method [39]. The intestine and liver sample supernatants were collected in China following the Nanjing Jiancheng Bioengineering Institute's instructions. They were separated by crushing 1 g of each sample in a homogenate medium, homogenizing, and centrifuging at 2500 rpm for 10 min at 4°C. The supernatant was stored at 4°C until the experiment. The Coomassie brilliant blue method and a commercial kit determined sample homogenate protein content [39]. After that, total antioxidant capacity (T-AOC) parameters, including T-AOC (A015-2-1), malondialdehyde (MDA) (A003-1), catalase (CAT) (A007-1-1), and superoxide dismutase (SOD) (A001-1) were detected following Nanjing Jiancheng Bioengineering Institute's instructions.

### 2.9. RNA Extraction, Reverse Transcription, and Real-Time Quantitative Polymerase Chain Reaction

According to the previous method [40], the fish samples were freeze-pulverized and imbedded in Trizol reagent (Vazyme Biotech Co., Ltd., China) to extract the total RNA. Total RNA integrity and concentration were determined using a 1.2% denaturing agarose gel and a NanoDrop spectrophotometer (Thermo Scientific, Nanodrop 2000 spectrophotometer). With the help of RNA-Free DNase, RNA was further cleaned of DNA impurities before being reverse transcribed into a single-stranded cDNA using the instructions provided (Biometra TRIO, analytikjena). This study used β-actin as internal reference gene for RT-PCR. The amplification volume was 20 μL and included 0.5 μL of primers, 10 μL of Takara TB Green Premix Ex TaqTM (Tli RNaseH Plus), 4 μL of cDNA product, and 5 μL of RNase-free water. The PCR cycling conditions were as follows: 95°C for 2 min, then 40 cycles of 95°C for 10 s, 59°C for 10 s, and 72°C for 20 s. A melting curve was used to assess the precision and sterility of all PCR products. RT-PCR was performed (FMR3, Real-time Quantitative Thermal Cycler, Vazyme), and relative gene expression levels were calculated using the 2−^ΔΔCT^ method [41]. The RT-PCR primer sequences used in this study are listed in Table 2.

### 2.10. Calculations and Statistical Analysis

  
$$\begin{array}{c} \text{Survival rate}\left(\text{SR},\%\right)=\left[\frac{\text{Final number of fish}}{\text{Initial number of fish}}\right]\times 100, \end{array}$$

  
$$\begin{array}{c} \text{Weight gain rate }\left(\text{WGR},\%\right)=\left[\frac{\text{Final body weight }-\text{Initial body weight}}{\text{Initial body weight}}\right]\times 100, \end{array}$$

  
$$\begin{array}{c} \text{Specific growth rate }\left(\text{SGR},\%\right)=\left[\frac{\text{Lnfinal body weight }-\text{Lninitial body weight}}{\text{Number of experimental days}}\right]\times 100, \end{array}$$

  
$$\begin{array}{c} \text{Hepatosomatic index }\left(\text{HSI},\%\right)=\left[\frac{\text{Liver weight}}{\text{body weight}}\right]\times 100, \end{array}$$

  
$$\begin{array}{c} \text{Viscerosomatic index }\left(\text{VSI},\%\right)=\left[\frac{\text{Viscera weight}}{\text{body weight}}\right]\times 100, \end{array}$$

  
$$\begin{array}{c} \text{Feed efficiency ratio }\left(\text{FER}\right)=\left[\frac{\text{Wet weight gain}}{\text{dry feed intake}}\right], \end{array}$$

  
$$\begin{array}{c} \text{Condition Factor }\left(\text{CF},\%\right)=\left[\frac{\text{Final weight}}{\text{body length}3}\right]\times 100. \end{array}$$

All statistical analyses were conducted using IBM SPSS 25.0. The data for each treatment were analyzed using one-way analysis of variance followed by Tukey's test for post hoc comparisons. The study results were presented as means ± SEM (standard error of the mean), with a significance level of *p* < 0.05. The graphic representations were generated using GraphPad Prism 9.0 (GraphPad Software Inc., USA).

## 3. Result

### 3.1. SR, Growth Performance and Proximate Composition Analysis in Fish Body

After the feeding trial, no significant differences were observed in SR among the different dietary treatment groups (Table 3). The final body weights (FBW) decreased significantly when the replacement of FO by DCSO exceeded 50% (*p* < 0.05). Consistent with FBW, the WGR and SGR exhibit the same pattern (*p*  < 0.05) when the replacement exceeds 50%. Meanwhile, the feed efficiency ratio (FER) showed a decrease pattern with the higher replacement of FO and exhibited a significant decline at a 100% replacement level. However, the VSI and HSI increased significantly (*p* < 0.05) after the replacement of FO by DCSO exceeded 50%. The proximate composition of the fish did not alter among juvenile large yellow croaker that fed with different diets (Table 4). Furthermore, the total lipid levels in the liver significantly elevated (*p* < 0.05) that corresponds to the higher replacement of FO by DCSO and reached statistical significance at replacement levels of 75% and 100% (Table 4). These results indicated that FO replacement by DCSO up to 50% did not negatively affect growth performance and feed utilization in large yellow croaker.

### 3.2. Fatty Acid Analysis of the Fish Tissues

In order to evaluate the effects of DCSO inclusion on fish tissue fatty acid composition, we analyzed the fatty acid profiles of experimental diets and fish tissues from liver and muscle. As indicated in Table 5, compared to FO, the DCSO exhibits the same features shared by the plant-based oil sources: abundance in *n*-6 PUFA and (mainly linoleic acid) scarcity in *n*-3 PUFA. Correspondingly, with the increasing replacement of FO by DCSO, the *n*-6 PUFA fatty acids gradually enriched while the *n*-3 PUFA were gradually missing in the diets. As a result, both liver and muscle exhibited similar patterns of fatty acid profiles exhibited in the diets, with accumulating *n*-3 PUFA and reducing of *n*-6 PUFA levels observed in these tissues (Tables 6, 7). Furthermore, these fatty acid profiles were altered in the tissues when the replacement of FO exceeded 50%. Moreover, the discrepancy of total SFA between the FO and DCSO in the diet was reflected in the tissues. The SFA elevated from FO fed fish group to DCSO inclusion groups (Table 6 and 7). Similarly, the alteration of SFA reached significant when the inclusion of DCSO exceeded 50% (*p* < 0.05). In DHA and EPA of DCSO25 and DCSO50 exposed significantly lower value from FO group while it was significantly higher from DCSO75 and DCSO100. Altogether, these results indicated that FO replacement by DCSO exceeding 50% could alter the fatty acid profiles of the fish tissues massively.

### 3.3. Serum Biochemical Indexes and Liver Function Analysis

To determine the effects of FO replacement by DCSO on liver functions, serum biochemical indexes, including TC, TG, LDL-C, ALT and AST were measured (Table 8). Compared to FO groups, the TC, TG and LDL-C were significantly increased (*p* < 0.05) when the inclusion of DCSO exceeded 50% (Table 8). Similarly, compared to FO groups, FO replacement exceeded 50% by DCSO, which significantly enhanced (*p* < 0.05) the enzyme activities of ALT and AST (Table 2). Among these, LDL-C and ALT are remarkably influenced by DCSO. HDL-C didn't reveal any notable change among different treatments. These results indicated that FO replacement exceeded 50% by DCSO significantly altered lipid contents in plasma and impaired the functions of fish liver.

### 3.4. Antioxidant Capacity

**Figure 1:**
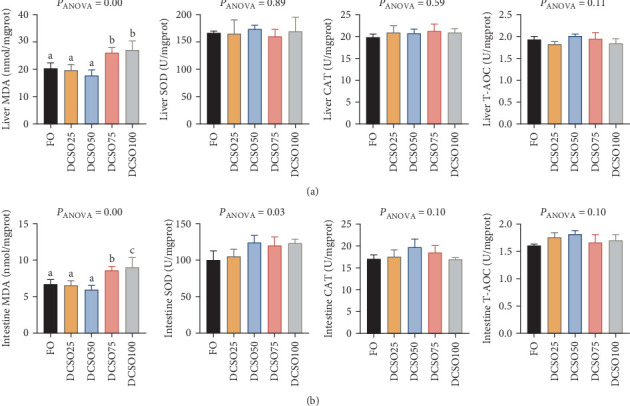
(A) concentration of malondialdehyde (MDA) and activities of superoxde dismutase (SOD), catalase (CAT), and total antioxidant capacity (T-AOC) in the liver (B) concentration of malondialdehyde (MDA) and activities of superoxide dismutase (SOD), catalase (CAT), and total antioxidant capacity (T-AOC) in the intestine. The data are expressed as means ± SEM. a, b, and c, mean values in each bar sharing the common superscripts letter are not significantly different as determined by Tukey's multiple range test (*p* < 0.05). ANOVA, analysis of variance; SEM, standard error of means (*n* = 3).

To further investigate the effects of FO replacement by DCSO on oxidative status of fish, antioxidant capacity including MDA, enzymes including SOD, T-AOC, and CAT from the liver and intestine of experimental fish were measured (Figure 1A and B). In both tissues, higher inclusion of DCSO did not alter the enzyme activities in SOD, T-AOC and CAT (Figure 1). However, compared to FO groups, FO replacement exceeded 50% by DCSO significantly increased (*p* < 0.05) the level of MDA in both the liver and intestine (Figure 1). This study demonstrated that the excess replacement of FO by DCSO would significantly increase the oxidative stress of the fish.

### 3.5. Enzyme Activity and Gene Expression Related to Lipid Metabolism

**Figure 2:**
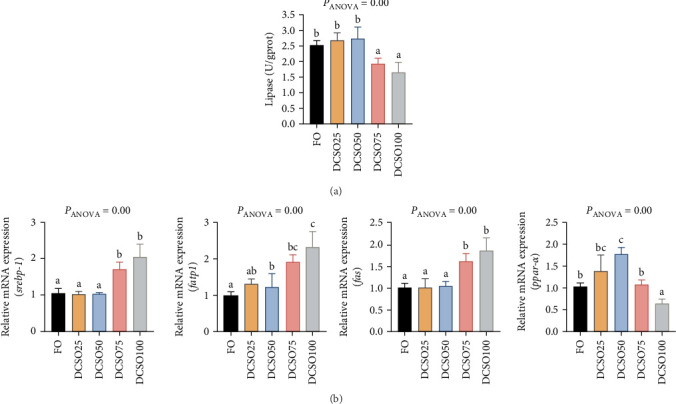
(A) activities of main digestive enzyme, Lipase, (B) expression of genes related to lipid metabolism in the liver of large yellow croaker: sterol-regulatory element-binding protein 1 (*srebp1*), fatty acid transport protein 1 (*fatp1*), fatty acid synthase (*fas*), and peroxisome proliferator-activated receptor α (*ppar-α*). The data are expressed as Mean ± SEM. a, b, and c, mean values in each bar sharing the common superscripts letter are not significantly different as determined by Tukey's multiple range test (*p* < 0.05). ANOVA, analysis of variance; SEM, standard error of means (*n* = 3).

To determine the effect of DCSO on lipid metabolism, the lipase activity from the intestine of the fish was examined. Compared to the FO groups, DCSO inclusion in the diets resulted an inhibiting effect on lipase activity, and 50% of FO replacement by DCSO could significantly decrease (*p* < 0.05) lipase activity (Figure 2A). However, the induction of genes that related to lipid synthesis, including sterol regulatory element-binding protein 1 (*srebp-1*), fatty acid synthase (*fas*) and fatty acid transport protein (*fatp1*), exhibited an decreasing pattern up to 50% replacement level but no significant difference (*p* < 0.05), but the replacement levels up to 50% or above could increase the expression levels (Figure 2B). Furthermore, unlike the lipid synthesis-related genes, the gene expression level of peroxisome proliferator-activated receptor α (*ppar-α*) revealed an opposite trend with the increasing inclusion of DCSO (Figure 2B), and an inclusion level of DCSO, higher than 50% in the diet could significantly inhibit the induction of *ppar-α* in the liver. These results indicated that more than 50% inclusion of DCSO increased lipid accumulation by aggravating lipid synthesis in the liver.

### 3.6. Expression of Genes Related to Inflammation

**Figure 3:**
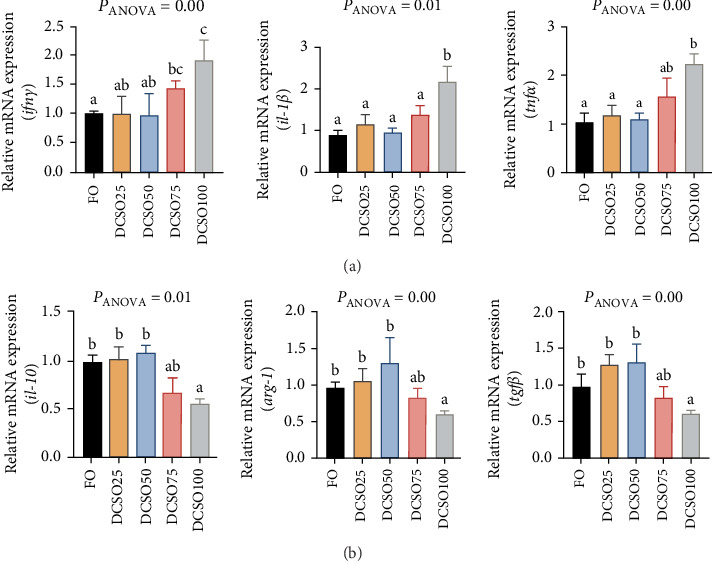
Expression of genes related to inflammation in the liver of large yellow croaker. (A) Pro- inflammatory genes: interferon γ (*ifnγ*), interleukin-1 b (*il-1 b*), and tumor necrosis factor α (*tnfα*); (B) anti-inflammatory genes: interleukin-10 (*il-10*), arginase-1 (*arg-1*) and transforming growth factor β (*tgfβ*). The data are expressed as means ± SEM. Mean values in each bar sharing the common superscripts letter are not significantly different as determined by Tukey's multiple range test (*p* < 0.05). ANOVA, analysis of variance; SEM, standard error of means (*n* = 3).

To evaluate the effect of FO replacement by DCSO on inflammation in liver, genes expression level that related to pro-inflammatory and anti-inflammatory were studied. Compared to FO groups, interleukin 1 β (*il-1β*), interferon γ (*ifnγ*) and tumor necrosis factor α (*tnfα*) exhibited a decreasing pattern up to 50% inclusion of DCSO but no remarkable difference. More than 50% inclusion of DCSO elevated the expression of *il-1β*, *ifnγ* and *tnfα* but only DCSO100 resulted significant difference (*p* < 0.05) (Figure 3A). However, inclusion of DCSO in the diet did not alter the expression level of *il-6* (Figure 3A). Moreover, Compared to FO groups, DCSO inclusion groups exhibited a decline pattern in inducting anti-inflammatory genes expression, and the interleukin 10 (*il-10*) and arginase 1 (*arg-1*) expression levels were significantly decreased (*p*  < 0.05) in DCSO100 groups (Figure 3B). Furthermore, the expression of transforming growth factor β (*tgfβ*) exhibited the same pattern, of which *tgfβ* expression decreased with the increasing addition of DCSO and reached notable reduction in groups DCSO75 and DCSO100 (*p* < 0.05). Altogether, these results suggested that inclusion of DCSO would activate the inflammation in the liver more than 75% inclusion level.

## 4. Discussion

In present study, we exploited the DCSO as an alternative for FO replacement in large yellow croaker juvenile in order to evaluate growth performance and nutritional effects of fish. The results of the present study indicated that FO replacement by DCSO up to 50% did not negatively affect growth performance and feed utilization in large yellow croaker. When the replacement of FO was higher than 50%, the detrimental effects on growth performance, fish physiological processes and health status were accumulated and amplified, which were consistent with the previous finding that an excessive amount of plant-based oil in the diet could ultimately impair the development and growth of large yellow croaker [34] and grass carp (*Ctenopharyngodon idella* [43]. In a previous study with black seabream (*Acanthopagrus schlegelii*), more than 60% replacement of FO by DCSO could reduce growth rate, feed efficiency and evoke inflammation in the liver [23]. However, our results demonstrated that including more than 50% of DCSO would not affects growth, feed efficiency and elicit inflammation in the liver.

The excess accumulation of lipids in the liver is a common physiological feature in the excessive inclusion of plant-based oil in aquafeeds, especially in marine carnivorous species, and negatively affects the growth performance of fish like juveniles *D. labrax* [44]. Plant-based oils are generally scarce in *n*-3 PUFA and abundant in *n*-6 PUFA. In this study, the fatty acid profiles of the experimental diets and target tissues from the fish were analyzed. The patterns of EPA and DHA in feed, liver, and muscle decreased significantly from FO group to DCSO treated group. However, the rate of decrease was lower than that in diets up to 50% replacement level, indicating that DHA and EPA tended to accumulate in the body to maintain the normal physiological function of the fish [17, 22]. The deposition of SFA exhibited same pattern with the higher inclusion of DCSO. In mammals, studies indicate that a ratio of *n*-6/*n*-3 PUFA in the diets could affect lipid metabolism by regulating *srebp-1* and *pparα* in other animals also, such as rat [45] and pigs [46], thus shifting the metabolism to anabolism by promoting fatty acids synthesis in fish [47] and inhibiting lipid degradation [48] in the liver. In present study, the low ratio of *n*-3/*n*-6 PUFA in DCSO evoked fatty acids synthesis and inhibited lipid degradation, which could partially explain the lipid accumulation in the liver. Furthermore, since marine fish species lack the synthetic capacity of *n*-3PUFA [49], the synthetic fatty acids (mainly SFAs) generated from the above processes were enriched and stuck in the liver, evidenced by the opposite deposition pattern of SFA in the liver. As a result, with the increase of DCSO inclusion, the discrepancy in fatty acid profiles in the high DCSO-containing diets would eventually aggregate the excess lipid accumulation (non n-3PUFA) in the liver.

Blood lipids can reflect lipid metabolism in the body. In current study, we observed that the replacement of FO by DCSO led to increase lipid contents, including T-CHO, LDL-C and total TG. The elevated plasma lipid content indicated that fatty acid biosynthesis activities were enhanced by alternating lipid resources in the experimental fish. Meanwhile,SFAs are known to cause fatty liver disease and impaired liver functions [18]. The enhanced fatty acids biosynthesis and excess accumulation of SFAs may contribute to the deterioration of liver function, especially in higher DCSO inclusion groups. ALT and AST are species and age-dependent, but increasing plasma ALT and AST diminish liver activities [18]. Compared to FO groups, the significantly aggregate activities of plasma ALT and AST were observed in DCSO100 groups, indicating that the full substitution of FO by DCSO could impair liver function. However, whether the SFAs generated from the biosynthesis in the liver or derived from the unbalanced profiles in the DCSO that evoked the pathological process of fatty liver is still an unanswered question.

Previous studies illustrated that the replacement of dietary FO with plant-based oils could diminish lipid peroxidation levels by reducing LC-PUFA levels to enhance antioxidant capacity [50]. In the current study, we demonstrated that the increasing substitution of FO by DCSO did not alter the activities of SOD, T-AOC and CAT in the liver, probably due to the high percentage of n-6PUFA (mainly linoleic acid) in the composition of DCSO fatty acid profiles. MDA is a key marker that reflects lipid peroxidation and oxidative stress [51]. Contradictory to a previous finding that the substitution of FO by vegetable oils led to MDA contents decreased in serum and liver in *Onychostoma macrolepis* [52], our results indicated that the concentration of MDA in the livers and intestines was elevated significantly when the inclusion of DCSO exceeded 50%. Although the impaired liver function and the excessive accumulation of lipids may exacerbate the oxidative capacities in the high DCSO inclusion groups, whether the increase of MDA is the cause or consequence of damage in liver function is still unknown. Future studies may focus on the underlying mechanisms of how unbalanced fatty acids deprave liver functions in marine fish species.

The dietary composition of fatty acids significantly impacts lipid metabolism, particularly in lipid synthesis and β-oxidation. *Srebp1*, *fatp1*, and *fas* are pivotal in hepatic lipid synthesis and are rate-limiting enzymes for fatty acid production [53]. This study found that fish-fed diets with elevated levels of DCSO exhibited significantly increased mRNA expression of the lipid synthesis gene. A substantial replacement of DCSO for FO resulted in a significant decrease in n-3 PUFAs in the diet, accompanied by a notable increase in SFAs, thereby diminishing the inhibitory effects in the liver [32]. In contrast, β-oxidation is a crucial mechanism of lipid metabolism in fish [54]. The pivotal energy metabolism gene, *ppar-α*, predominantly expressed in the hepatic tissue of fish, regulates several crucial enzymes involved in fatty acid β-oxidation [32]. This study demonstrated that a fish-fed diet comprising over 50% DCSO resulted in markedly reduced mRNA expression of *ppar-α*. A prior study involving Nile tilapia resulted that reduced expression of *ppar-α* may diminish lipid degradation, akin to mammals [55]. Consequently, it was determined that the primary factors contributing to the elevated lipid content in the liver are likely the inhibition of *ppar-α* and the activation of *srebp-1*, *fatp1*, and *fas*, which expedite the lipid accumulation process in the liver.

Pro-inflammatory genes are crucial in initiating and promoting inflammatory responses [56]. Upregulation of these genes typically indicates the presence of inflammation and immune activation in the liver [57]. Elevated expression of these pro-inflammatory genes can negatively impact growth performance, as diversifying energy and resources towards the immune response can compromise growth and feed utilization efficiency [56, 57]. Additionally, the upregulation of inflammatory cytokines can disrupt normal lipid metabolism, leading to abnormal fatty acid profiles and excessive lipid deposition in the liver [58]. Anti-inflammatory genes promote the production of anti-inflammatory mediators and suppress the activity of pro-inflammatory pathways [59]. Upregulation of anti-inflammatory genes can indicate a balanced immune response and help maintain normal liver function [60], crucial for supporting optimal growth performance and healthy lipid metabolism [61]. The balance between pro- and anti-inflammatory gene expressions in the liver can provide insights into the overall health and physiological status in response to the dietary treatments [62, 63]. In the present study, Pro-inflammatory genes (*ifnγ*, *il-1β*, and *tnfα*) significantly elevated their expression in DCSO75 and DCSO100. In contrast, Anti-inflammatory genes (*il-10*, *arg-1*, and *tgfβ*) were significantly deprived of their expression in DCSO75 and DCSO100. These inflammatory responses may impair liver function, which is essential for overall fish health, potentially affecting growth rates and feed efficiency. Dietary DCSO at an appropriate level has been claimed to have numerous beneficial health effects.

The importance of these findings resides in the capacity of DCSO to influence fish health and feed formulation methodologies. Comprehending these alterations in gene expression can aid in evaluating the nutritional value and potential risks of utilizing DCSO as an alternative lipid source in aquaculture.

## 5. Conclusion

Present study assessed the DCSO as an alternative oil source for FO. This study evaluated the effects of FO replacement with DCSO on growth performance, proximate composition, lipid metabolism, and liver function of large yellow croaker juvenile. Results indicated that FO replacement by DCSO up to 50% did not negatively affect growth performance and feed utilization in large yellow croaker. Moreover, a high amount of replacement of FO will lead to adverse effects, including compromised growth performance, the occurrence of fatty liver as well as impaired liver function. In brief, these findings systematically evaluate DCSO as an alternative to FO and offer their optimal proportion when considering optimal lipid sources.

## Figures and Tables

**Table 1:** Formulations and proximate composition of experimental diets fed to juvenile large yellow croaker.

Ingredients (g/kg)	FO	DCSO25	DCSO50	DCSO75	DCSO100
White fish meal^a^	310.00	310.00	310.00	310.00	310.00
Krill meal^b^	10.00	10.00	10.00	10.00	10.00
Soybean meal^c^	255.50	255.50	255.50	255.50	255.50
Bread flour^d^	294.00	294.00	294.00	294.00	294.00
Fish oil (FO)	70.00	52.50	35.00	17.50	0.00
Degossypolized Cottonseed oil (DCSO)^g^	**0.00**	**17.50**	**35.00**	**52.50**	**70.00**
Soybean lecithin	20.00	20.00	20.00	20.00	20.00
Choline chloride	2.00	2.00	2.00	2.00	2.00
Vitamin C phosphate	0.50	0.50	0.50	0.50	0.50
Ca (H_2_PO_4_)_2_	20.00	20.00	20.00	20.00	20.00
Vitamin premix^e^	2.00	2.00	2.00	2.00	2.00
Mineral premix^f^	10.00	10.00	10.00	10.00	10.00
Mold inhibitor	0.50	0.50	0.50	0.50	0.50
Attractant (glycine & betaine)	5.00	5.00	5.00	5.00	5.00
Aantioxidant (TBHQ)	0.50	0.50	0.50	0.50	0.50
Total	1000	1000	1000	1000	1000
Proximate composition (dry matter basis)					
Moisture	115.60	125.60	124.90	110.00	110.20
Crude protein	418.20	412.90	411.00	412.30	410.60
Crude lipid	133.20	127.20	130.30	134.80	126.90
Crude ash	56.70	53.40	50.20	59.80	54.50
Gross energy (MJ/kg)	18.60	18.35	18.14	18.65	18.66

*Note*: The ingredients were sourced from Great Seven Biotechnology Co., Ltd., Shandong, China. Degossypolized Cottonseed oil was bought from Xinjiang Herun Jinlan Biotechnology Co. Ltd.

^a,b,c,d^White fish meal (71.22% crude protein, 11.26% crude lipid); krill meal (52.76% crude protein, 12.10% crude lipid); soybean meal (46.68% crude protein, 0.33% crude lipid); bread flour (17.39% crude protein, 0.61% crude lipid).

^e^Vitamin premix (mg/kg diet): retinal acetate, 32; alpha-tocopherol, 240; menadione, 10; thiamin, 25; pyridoxine HCl, 20; vitamin B12, 10; riboflavin, 45; pantothenic acid, 60; cholecalciferol, 5; folic acid, 20; niacin 200; biotin, 60; inositol, 800; microcrystalline cellulose, 13473. Vitamin C was supplied in the form of vitamin C polyphosphate.

^f^Mineral premix (mg/kg diet): MgSO_4_ · 7H_2_O, 1200; FeSO_4_ · H_2_O, 80; ZnSO_4_ · H_2_O, 50; CuSO_4_ · 5H_2_O, 10; MnSO_4_ · H_2_O, 45; CoCl_2_ · 6H_2_O, 50; Na_2_SeO_3_, 20; H_2_CaIO_4_, 60; zeolite powder, 13485

^g^Degossypolized Cottonseed oil (100 g oil): (3700 KJ energy; 0 g protein; 99.99 g fat; 0 g carbohydrate; 0 g sodium). Edible degossypolized cottonseed oil with no gossypol (less than 1μg/kg) gossypol.

**Table 2:** Sequences of the RT-PCR primers used in this study.

Target genes	Forward (5ʹ-3ʹ)	Reverse (5ʹ-3ʹ)	References
*srebp1*	TCTCCTTGCAGTCTGAGCCAAC	TCAGCCCTTGGATATGAGCCT	KP342262
*fatp1*	CAACCAGCAGGACCCATTACG	CATCCATCACCAGCACATCACC	KM593124
*fas*	CAGCCACAGTGAGGTCATCC	TGAGGACATTGAGCCAGACAC	JX456351
*ppar-α*	GTCAAGCAGATCCACGAAGCC	TGGTCTTTCCAGTGAGTATGAGCC	[42]
*ifnγ*	TCAGACCTCCGCACCATCA	GCAACCATTGTAACGCCACTTA	KM501500
*il-1β*	CATAGGGATGGGGACAACGA	AGGGGACGGACACAAGGGTA	KJ459927
*tnfα*	ACACCTCTCAGCCACAGGAT	CCGTGTCCCACTCCATAGTT	EF165623
*il-10*	AGTCGGTTACTTTCTGTGGTG	TGTATGACGCAATATGGTCTG	XM010738826
*arg1*	AACCACCCGCAGGATTACG	AAACTCACTGGCATCACCTCA	XM019269015
*tgfβ*	AGCAACCACCGTACATCCTG	AGGTATCCCGTTGGCTTGTG	XM027280465
*β-actin*	GACCTGACAGACTACCTCATG	AGTTGAAGGTGGTCTCGTGGA	GU584189

Abbreviations: β-actin, β-Actin; arg-1, arginase-1; fas, fatty acid synthase; fatp1, fatty acid transport protein 1; ifn*γ*, interferon γ; il-1β, interleukin-1β; il-10, B interleukin-10; ppar-*α*, peroxisome proliferator-activated receptor α; srebp1, Sterol-regulatory element-binding protein 1; tnf*α*, tumor necrosis factor α; tgfβ, transforming growth factor β.

**Table 3:** Survival, growth, feed utilization, and morphometric parameters of large yellow croaker fed different percentages of degossypolized cottonseed oil.

Treatments Index	FO	DCSO25	DCSO50	DCSO75	DCSO100	*P*_ANOVA_
FBW (g)	32.95 ± 1.34^c^	33.43 ± 0.71^c^	34.55 ± 1.26^c^	27.9 ± 2.41^b^	23.98 ± 1.79^a^	0.00
Survival (%)	82.22 ± 12.50	80.55 ± 2.54	83.33 ± 13.22	75 ± 14.52	74.55 ± 6.73	0.82
WGR (%)	297.97 ± 16.15^c^	303.74 ± 8.57^c^	317.31 ± 15.21^c^	236.96 ± 19.93^b^	189.69 ± 21.66^a^	0.00
SGR (%/d)	1.94 ± 0.07^a^	1.97 ± 0.03^a^	2.01 ± 0.05^a^	1.70 ± 0.12^b^	1.49 ± 0.1^c^	0.00
FER	0.75 ± 0.13^b^	0.67 ± 0.03^ab^	0.64 ± 0.03^ab^	0.57 ± 0.05^ab^	0.53 ± 0.06^a^	0.02
VSI (%)	5.15 ± 0.15^a^	6.28 ± 0.51^a^	6.28 ± 0.09^a^	7.95 ± 0.47^b^	8.03 ± 0.73^b^	0.00
HSI (%)	1.87 ± 0.05^a^	2.03 ± 0.3^a^	2.11 ± 0.48^a^	2.97 ± 0.48^b^	2.90 ± 1.18^b^	0.00
CF	1.11 ± 0.14	1.19 ± 0.01	1.13 ± 0.07	1.05 ± 0.03	1.03 ± 0.07	0.20

*Note*: The data are expressed as Mean ± S.E.M. The data in each row with common superscript letters shows no significant differences as determined by Tukey's multiple range test (*p* < 0.05).

Abbreviations: ANOVA, analysis of variance; CF, Condition factor; FBW, Final body weight; FER, Feed efficiency ratio; HIS, Hepatosomatic index; SEM, standard error of means (*n* = 3); SGR, Specific growth rate; VSI, Viscerosomatic index index; WGR, Weight gain rate.

**Table 4:** Body composition (dry weight %) of large yellow croaker fed diets with different percentages of degossypolized cottonseed oil.

Parameters	FO	DCSO25	DCSO50	DCSO75	DCSO100	*P*_ANOVA_
Moisture	72.33 ± 2.33	74.79 ± 2.54	74.31 ± 0.15	74.54 ± 0.45	74.99 ± 1.27	0.35
Crude protein	56.99 ± 4.31	57.52 ± 0.74	57.945 ± 1.94	57.46 ± 2.71	58.598 ± 2.07	0.98
Crude lipid	29.39 ± 3.67	29.69 ± 2.53	28.95 ± 2.83	28.56 ± 2.90	31.33 ± 1.68	0.78
Crude ash	11.35 ± 0.73	11.48 ± 0.85	10.84 ± 0.77	11.22 ± 0.96	11.13 ± 0.14	0.93
Liver moisture	58.65 ± 2.89	60.89 ± 3.89	61.29 ± 3.98	62.78 ± 4.45	61.78 ± 4.56	0.13
Liver crude lipid	59.25 ± 3.34^a^	61.54 ± 2.09^ab^	60.43 ± 2.01^ab^	67.25 ± 2.34^bc^	72.72 ± 3.41^c^	0.00

*Note*: The data are expressed as means ± S.E.M. The data in each row with common superscript letters shows no significant differences as determined by Tukey's multiple range test (*p* < 0.05).

Abbreviations: ANOVA, analysis of variance; SEM, standard error of means (*n* = 3).

**Table 5:** Fatty acid profiles of the experimental diets (% total fatty acids).

Fatty acid	FO	DCSO	Degossypolized cottonseed oil replacement levels (%)				
			FO	DCSO25	DCSO50	DCSO75	DCSO100
C14:0 Mystric acid	6.00	0.80	8.35	4.45	2.88	2.30	1.65
C16:0 Palmitic acid	20.20	23.40	20.24	20.89	21.34	23.44	24.89
C18:0 Stearic acid	4.10	2.50	8.67	6.56	5.66	4.34	4.01
C20:0	0.80	0.40	0.91	0.76	0.72	0.67	0.66
*Σ*SFA^a^	31.10	27.10	38.17	32.66	30.6	30.75	31.21
C16:1*n*–9	5.10	0.80	8.33	7.28	6.13	6.07	5.67
C18:1*n*–9 oleic acid	12.65	15.61	6.31	6.77	8.55	9.60	10.33
*Σ*MUFA^b^	17.75	16.41	14.64	14.05	14.68	15.67	16.00
C18:2*n*–6 Linoleic acid	3.20	55.00	6.23	18.56	23.45	28.8	33.78
C20:4*n*–6 ARA	0.81	nd	0.60	0.51	0.37	0.33	0.25
*Σ n−*6PUFA^c^	4.01	55.00	6.83	19.07	23.82	29.13	34.03
C18:3*n*–3 LNA	1.30	0.40	1.40	1.23	1.00	0.88	0.76
C20:5*n*–3 EPA	8.60	nd	8.56	6.34	4.56	3.32	1.34
C22:6*n*–3 DHA	15.40	nd	13.55	9.65	8.89	4.34	2.98
*Σ n−*3PUFA^d^	25.30	0.40	23.51	17.22	14.45	8.54	5.08
*n−*3/*n−*6PUFA	—		3.44	0.90	0.61	0.29	0.15

^a^SFA, saturated fatty acids.

^b^MUFA, monounsaturated fatty acids.

^c^ *n−*6 PUFA, *n−*6 polyunsaturated fatty acids.

^d^ *n−*3 PUFA, *n−*3 polyunsaturated fatty acids.

**Table 6:** Fatty acid composition (% total fatty acids) in the liver of large yellow croaker fed diets with different graded levels of degossypolized cottonseed oil.

Fatty acids (%)	Degossypolized cottonseed oil replacement levels (%)					*P*_ANOVA_
	FO	DCSO25	DCSO50	DCSO75	DCSO100	
C14:0	1.49 ± 0.43^b^	1.41 ± 0.43^ab^	1.33 ± 0.06^ab^	1.10 ± 0.07^ab^	0.85 ± 0.05^a^	0.04
C16:0	22.19 ± 0.1.91^a^	23.52 ± 0.94^ab^	24.20 ± 0.85^ab^	26.13 ± 1.20^ab^	25.83 ± 1.86^b^	0.04
C18:0	1.80 ± 0.2^a^	1.91 ± 0.06^a^	2.19 ± 0.18^a^	2.40 ± 0.14^a^	3.70 ± 0.58^b^	0.00
C20:0	0.22 ± 0.03^a^	0.26 ± 0.02^ab^	0.30 ± 0.01^bc^	0.34 ± 0.02^cd^	0.39 ± 0.02^d^	0.00
C22:0	0.48 ± 0.07^a^	0.70 ± 0.05^b^	0.66 ± 0.06^b^	0.80 ± 0.07^b^	0.76 ± 0.04^b^	0.00
*Σ*SFA	26.19 ± 2.00^a^	27.81 ± 1.10^ab^	28.69 ± 1.09^ab^	30.79 ± 0.98^b^	31.55 ± 1.59^b^	0.01
C16:1*n*–9	2.48 ± 0.16	2.82 ± 0.05	2.50±0.14 b	2.31 ± 0.15	2.48 ± 0.28	0.15
C18:1*n*–9	11.26 ± 1.05^a^	12.85 ± 0.34^ab^	16.00 ± 0.66^bc^	18.13 ± 0.49^c^	22.53 ± 2.80^d^	0.00
*Σ*MUFA	13.75 ± 1.11^a^	15.68 ± 0.3^a^	18.50 ± 0.54^ab^	20.44 ± 0.39^b^	24.83 ± 2.31^b^	0.00
C18:2*n*–6	20.03 ± 1.66^a^	22.18 ± 1.61^ab^	21.85 ± 1.03^ab^	25.24 ± 0.98^bc^	25.74 ± 1.38^c^	0.00
C20:4*n*–6	0.42 ± 0.05	0.34 ± 0.06	0.35 ± 0.04	0.30 ± 0.04	0.35 ± 0.05	0.05
*Σ n*-6PUFA	20.46 ± 1.69^a^	22.53 ± 1.55^ab^	22.20 ± 0.99^a^	25.58 ± 1.00^b^	26.04 ± 1.40^b^	0.00
C18:3*n*–3	1.85 ± 0.06	1.98 ± 0.14	1.91 ± 0.17	1.93 ± 0.06	1.80 ± 0.05	0.41
C20:5*n*–3 EPA	5.70 ± 0.23^d^	5.19 ± 0.16^cd^	4.69 ± 0.15^c^	2.53 ± 0.22^ab^	1.76 ± 0.23^a^	0.00
C22:6*n*–3 DHA	6.43 ± 0.39^c^	6.07 ± 0.16^c^	5.52 ± 0.28^b^	2.56 ± 0.31^a^	1.83 ± 0.14^a^	0.00
*Σ n−*3PUFA	14 ± 0.47^c^	13.24 ± 0.30^c^	12.12 ± 0.20^c^	7.03 ± 0.59^b^	5.40 ± 0.42^a^	0.00
*n−*3/*n−*6PUFA	0.69 ± 0.05^c^	0.59 ± 0.04^b^	0.55 ± 0.03^b^	0.28 ± 0.03^a^	0.21 ± 0.02^a^	0.00

*Note*: The data are expressed as means ± S.E.M. The data in each row with common superscript letters shows no significant differences as determined by Tukey's multiple range test (*p* < 0.05).

Abbreviations: ANOVA, analysis of variance; SEM, standard error of means (*n* = 3).

**Table 7:** Fatty acid composition (% total fatty acids) in the muscle of large yellow croaker fed diets with different graded levels of degossypolized cottonseed oil.

Fatty acids (%)	Degossypolized cottonseed oil replacement levels (%)					*P*_ANOVA_
	FO	DCSO25	DCSO50	DCSO75	DCSO100	
C14:0	1.65 ± 0.0.20^b^	1.38 ± 0.0.27^ab^	1.20 ± 0.1^ab^	1.12 ± 0.15^a^	0.99 ± 0.16^a^	0.01
C16:0	21.34 ± 1.17^a^	23.42 ± 0.56^ab^	23.80 ± 1.41^ab^	26.26 ± 1.52^b^	27.52 ± 0.0.83^c^	0.04
C18:0	4.26 ± 0.35	4.43 ± 0.5	3.88 ± 0.55	4.40 ± 0.0.52	4.23 ± 0.46	0.50
C20:0	0.44 ± 0.12	0.36 ± 0.02	0.36 ± 0.07	0.53 ± 0.12	0.45 ± 0.11	0.01
C22:0	0.67 ± 0.10	0.72 ± 0.07	0.64 ± 0.13	0.76 ± 0.11	0.78 ± 0.03	0.15
*Σ*SFA	28.38 ± 1.33^a^	30.31 ± 0.83^a^	29.89 ± 1.05^ab^	33.06 ± 1.23^bc^	33.89 ± 0.49^c^	0.00
C16:1*n*–9	4.86 ± 0.55^a^	4.37 ± 0.46^ab^	3.99 ± 0.37^bc^	3.45 ± 0.40^bc^	2.79 ± 0.31^c^	0.00
C18:1*n*–9	14.86 ± 2.17^a^	15.86 ± 2.10^a^	18.85 ± 1.39^ab^	22.22 ± 1.25^b^	23.11 ± 1.49^b^	0.00
*Σ*MUFA	19.72 ± 2.44^a^	20.24 ± 2.52^a^	22.85 ± 1.68^ab^	25.67 ± 1.23^b^	25.91 ± 1.24^b^	0.00
C18:2*n*–6	14.48 ± 2.11^a^	17.67 ± 1.42^ab^	20.15 ± 1.68^b^	26.11 ± 2.13^c^	27.34 ± 2.74^c^	0.00
C20:4*n*–6	0.96 ± 0.08^d^	0.78 ± 0.11^cd^	0.67 ± 0.10^bc^	0.46 ± 0.0.86^ab^	0.34 ± 0.06^a^	0.05
*Σ n−*6PUFA	15.45 ± 2.06^a^	18.45 ± 1.31^b^	20.82 ± 1.72^b^	26.58 ± 2.22^c^	27.67 ± 2.69^c^	0.00
C18:3*n*–3	2.60 ± 0.35^b^	3.52 ± 0.28^ab^	3.45 ± 0.44^ab^	2.52 ± 0.54^ab^	2.30 ± 0.51^a^	0.04
C20:5*n*–3 EPA	6.81 ± 0.49^c^	5.53 ± 0.71^bc^	4.88 ± 0.52^b^	2.99 ± 0.64^a^	2.21 ± 0.59^a^	0.00
C22:6*n*–3 DHA	7.14 ± 0.86^c^	5.22 ± 0.28^b^	5.14 ± 0.75^b^	2.42 ± 0.49^a^	1.97 ± 0.45^a^	0.00
*Σ n−*3PUFA	16.56 ± 0.63^c^	14.27 ± 0.73^b^	13.47 ± 1.33^b^	7.94 ± 0.55^a^	6.49 ± 1.09^a^	0.00
*n−*3/*n−*6PUFA	1.08 ± 0.13^c^	0.78 ± 0.09^b^	0.65 ± 0.07^b^	0.30 ± 0.02^a^	0.23 ± 0.02^a^	0.01

*Note*: The data are expressed as means ± S.E.M. The data in each row with common superscript letters shows no significant differences as determined by Tukey's multiple range test (*p* < 0.05).

Abbreviations: ANOVA, analysis of variance; SEM, standard error of means (*n* = 3).

**Table 8:** Serum biochemical indexes and enzyme activities of large yellow croaker fed diets with different levels of degossypolized cottonseed oil (Means ± SEM).

Plasma biochemical Indexes	FO	DCSO25	DCSO50	DCSO75	DCSO100	*P*_ANOVA_
T-CHO (mmol/L)	2.29 ± 0.33^a^	2.66 ± 0.15^b^	3.18 ± 0.28^bc^	3.49±0.44 b^c^	3.71 ± 0.38^c^	0.00
TG (mmol/L)	3.18 ± 0.15^a^	3.40 ± 0.41^a^	3.49 ± 0.24^a^	4.39 ± 0.19^b^	4.91 ± 0.44^b^	0.00
HDL-C (mmol/L)	0.82 ± 0.13	0.78 ± 0.09	0.82 ± 0.10	0.69 ± 0.05	0.65 ± 0.07	0.15
LDL-C (mmol/L)	0.57 ± 0.04^a^	0.64 ± 0.01^a^	0.64 ± 0.04^a^	0.86 ± 0.02^b^	0.95 ± 0.07^b^	0.00
AST (U/L)	4.41 ± 0.99^a^	4.56 ± 0.72^ab^	4.82 ± 0.93^b^	6.54 ± 0.28^bc^	6.68 ± 0.53^c^	0.01
ALT (U/L)	51.05 ± 4.11^a^	49.43 ± 2.81^a^	48.31 ± 2.60^a^	53.50 ± 2.35^ab^	59.11 ± 1.85^b^	0.01

*Note*: The data are expressed as means ± S.E.M. The data in each row with common superscript letters shows no significant differences as determined by Tukey's multiple range test (*p* < 0.05).

Abbreviations: ALT, alanine transaminase; ANOVA, analysis of variance; AST, aspartate aminotransferase; HDL-C, high-density lipoprotein cholesterol; LDL-C, low-density lipoprotein cholesterol; SEM, standard error of means (n = 3); T-CHO, total cholesterol; TG, total triglyceride.

## Data Availability

Data will be made available upon request.

## Nomenclature

*Arg1*: Arginase-1 (involved in the metabolism of L-arginine to L-ornithine and urea)
CAT: Catalase (neutralizes hydrogen peroxide within cells)
cDNA: Complementary deoxyribonucleic acid
DCSO: Degossypolized cottonseed oil
DHA: Docosahexaenoic acid
EPA: Eicosapentaenoic acid
*fas*: Fatty acid synthase (plays a crucial role in de novo fatty acid synthesis)
*fatp1*: Fatty acid transport protein 1
FBW: Final body weight
FER: Feed efficiency ratio
FI: Feed intake
FO: Fish oil
*fxr*: Farnesoid X receptor
HDL-C: High-density lipoprotein cholesterol
HIS: Hepatosomatic index
HUFA: Highly unsaturated fatty acid
*il-1β*: Interleukin-1β (pro-inflammatory cytokine involved in initiating and regulating the inflammatory response)
*il-6*: Interleukin-6 (participates in the acute phase response by promoting the production of acute-phase proteins)
*il-10*: Interleukin-10 (anti-inflammatory cytokine which helps in regulating immune responses and maintaining immune homeostasis)
*ifn-γ*: Interferon-γ (involved in mediating immune responses against viral, bacterial, and parasitic infections)
LDL-C: Low-density lipoprotein cholesterol
LNA: Linoleic acid
LYC: Large yellow croaker
mRNA: Messenger ribonucleic acid
MUFA: Monounsaturated fatty acid
*pparα*: Peroxisome proliferator-activated receptor α (induces the expression of genes involved in fatty acid catabolism)
PUFA: Polyunsaturated fatty acid
RT-qPCR: Real-time quantitative polymerase chain reaction
SFA: Saturated fatty acid
SGR: Specific growth rate
*sod*: Superoxide dismutase (catalyze the dismutation of superoxide radicals into oxygen and hydrogen peroxide)
*srebp-1*: Sterol regulatory element-binding protein-1 (plays a crucial role in regulating lipid metabolism)
SR: Survival rate
T-AOC: Total antioxidant capacity
T-CHO: Total cholesterol
*tnf-α*: Tumor necrosis factor-alpha (plays a crucial role in the regulation of immune responses, inflammation)
VSI: Viscerosomatic index
WGR: Weight gain rate.
